# Autophagy activation in COL6 myopathic patients by a low-protein-diet pilot trial

**DOI:** 10.1080/15548627.2016.1231279

**Published:** 2016-09-22

**Authors:** Silvia Castagnaro, Camilla Pellegrini, Massimo Pellegrini, Martina Chrisam, Patrizia Sabatelli, Silvia Toni, Paolo Grumati, Claudio Ripamonti, Loredana Pratelli, Nadir M. Maraldi, Daniela Cocchi, Valeria Righi, Cesare Faldini, Marco Sandri, Paolo Bonaldo, Luciano Merlini

**Affiliations:** a Department of Molecular Medicine, University of Padova, Padova, Italy; b Laboratory of Musculoskeletal Cell Biology, Rizzoli Orthopedic Institute, Bologna, Italy; c Department of Diagnostic, Clinical and Public Health Medicine, University of Modena and Reggio Emilia, Modena, Italy; d Institute of Molecular Genetics, CNR National Research Council of Italy, Rizzoli Orthopedic Institute, Bologna, Italy; e Institute of Biochemistry II, Goethe University School of Medicine, Frankfurt am Main, Germany; f Department of Medicine and Rheumatology, University of Bologna, Bologna, Italy; g Clinical Pathology Unit, Rizzoli Orthopedic Institute, Bologna, Italy; h Department of Statistical Sciences, University of Bologna, Bologna, Italy; i Department of Life Quality Studies, Campus Rimini, University of Bologna, Bologna, Italy; j Department of Orthopedics, University of Bologna, Bologna, Italy; k Department of Biomedical Sciences, University of Padova, Padova, Italy; l Venetian Institute of Molecular Medicine, Padova, Italy; m CRIBI Biotechnology Center, University of Padova, Padova, Italy

**Keywords:** autophagy, Bethlem myopathy, clinical trial, collagen VI, low-protein diet, muscular dystrophies, Ullrich congenital muscular dystrophy

## Abstract

A pilot clinical trial based on nutritional modulation was designed to assess the efficacy of a one-year low-protein diet in activating autophagy in skeletal muscle of patients affected by COL6/collagen VI-related myopathies. Ullrich congenital muscular dystrophy and Bethlem myopathy are rare inherited muscle disorders caused by mutations of *COL6* genes and for which no cure is yet available. Studies in *col6* null mice revealed that myofiber degeneration involves autophagy defects and that forced activation of autophagy results in the amelioration of muscle pathology. Seven adult patients affected by COL6 myopathies underwent a controlled low-protein diet for 12 mo and we evaluated the presence of autophagosomes and the mRNA and protein levels for BECN1/Beclin 1 and MAP1LC3B/LC3B in muscle biopsies and blood leukocytes. Safety measures were assessed, including muscle strength, motor and respiratory function, and metabolic parameters. After one y of low-protein diet, autophagic markers were increased in skeletal muscle and blood leukocytes of patients. The treatment was safe as shown by preservation of lean:fat percentage of body composition, muscle strength and function. Moreover, the decreased incidence of myofiber apoptosis indicated benefits in muscle homeostasis, and the metabolic changes pointed at improved mitochondrial function. These data provide evidence that a low-protein diet is able to activate autophagy and is safe and tolerable in patients with COL6 myopathies, pointing at autophagy activation as a potential target for therapeutic applications. In addition, our findings indicate that blood leukocytes are a promising noninvasive tool for monitoring autophagy activation in patients.

## Introduction

COL6/collagen VI is a major extracellular matrix protein of skeletal muscle endomysium. Mutations of *COL6* genes (*COL6A1, COL6A2*, and *COL6A3*) cause COL6-related myopathies,^1^ a group of rare inherited muscle diseases that include Bethlem myopathy (BM) and Ullrich congenital muscular dystrophy (UCMD), as well as the limb girdle and myosclerosis myopathy variants.^2,3^ BM is characterized by axial and proximal muscle weakness,^4,5^ together with contractures of the interphalangeal joints of the last 4 fingers.^6^ BM is usually mild and sometimes slowly progressive. Respiratory failure is part of the clinical spectrum and can occur in ambulatory patients.^7^ UCMD is a severe congenital muscular dystrophy characterized by early onset, generalized muscle wasting and weakness, proximal joint contractures and distal joint hyperflexibility. Independent walking is rarely achieved or maintained in UCMD children, who also suffer from early and progressive respiratory failure.^1,8^ There is currently no cure for BM or UCMD.^9^

We previously showed that cyclosporin A is capable of rescuing muscle alterations associated with COL6 deficiency in *col6a1* null mice^10^ and in cells of BM or UCMD patients.^11,12^ Although clinical studies indicated that cyclosporin A may be helpful in slowing disease progression by correcting the mitochondrial dysfunction,^13,14^ the well-known immunosuppressive activity of cyclosporin A is a major hurdle and hinders its long-term use especially in pediatric patients. Recent studies in *col6a1* null mice and patients' biopsies provided novel insights into the molecular mechanisms underlying COL6-related myopathies. These studies showed that a failure of the autophagy machinery plays a major pathogenic role in muscle wasting and weakness.^15^ Autophagy is an evolutionarily conserved self-degradative process that is essential for retrieving nutrients during fasting and for the removal of harmful or damaged cellular components.^16,17^ In skeletal muscles of *col6a1* null mice, the impairment of autophagosome formation triggers the accumulation of dysfunctional mitochondria and aberrant organelles in myofibers, leading to myofiber apoptosis and myopathy.^15,18^ Notably, restoration of a proper autophagic flux by genetic, pharmacological or nutritional approaches rescues the myopathic phenotype of *col6a1* null mice.^15,19,20^ In particular, feeding *col6a1* null mice for one mo with a low-protein diet (LPD) leads to a dramatic recovery of the structural and functional muscle defects.^15^

Here we report the results of a pilot clinical study with a LPD carried out for 12 mo in 7 adult subjects with genetically characterized COL6 mutations (6 BM and 1 UCMD). The primary endpoints of this nutritional approach were successfully achieved, as the safety of the treatment was demonstrated and patients' muscles displayed increased levels of the autophagic markers BECN1 and MAP1LC3B/LC3B after the treatment. Moreover, and more importantly, this biological response was paralleled by a lack of disease progression and an improvement of some functional and metabolic parameters. Notably, the apoptosis incidence in muscle biopsies was significantly decreased after one y of LPD. These findings indicate that nutritional approaches may be effectively used for activating autophagy in patients affected by COL6-related myopathies and point at autophagy activation as a valuable therapeutic target for improving muscle health in these patients. In more general terms, our data provide a rationale for novel treatment opportunities in a growing number of muscle and nonmuscle pathologies associated with autophagy impairment.

## Results

The study included 8 patients. Five women and 3 men were recruited following the enrollment criteria described below. One participant left the trial soon after starting the LPD because of food intolerance; this patient subsequently had a serious traffic accident and was unable to participate in further evaluations. The patients' genetic and clinical data are summarized in Table 1. The study flow is described in Table S1.

**Table 1.** Description, clinical features and genetic characterization of the 7 patients with COL6-related myopathies that participated in the LPD trial.

Patient	Age/Gender	Diagnosis	Clinical features	Mutation
1	48/W	BM	Unable to run age 6 y, DL, FC, severe RI, A	*COL6A2* exon 6 c.802 G>A het; (p.Gly268Ser)^38^
2	36/M	BM	Unable to run age 18 y, FC, mild RI, A	*COL6A2* intron 25 c.1970-3 C>A het; (p.Thr656_Ala698del)^38^
3	19/W	UCMD	Floppy at birth, CHD, DL, FC, SC, MV age 11 yr, NA (walked 20 mo-6 y)	*COL6A1* exon8-intron 8 c.798_804+8del 15 het.; (p.Pro254_Glu268 del)^39^
4	27/M	BM	Clubfoot, mild distal limb weakness age 6 y, FC, normal respiratory function, A	*COL6A3* exon 11 c.4928 T>G het; (p.Leu1643Arg)^38^
5	28/M	BM	Clubfoot, never able to run, diffuse contractures, FC, moderate RI, A	*COL6A2* exon 26 c.2098 G>A; (p.Gly700Ser)^40^
6	41/W	BM	First steps at 2 y, never able to run, DL, FC, moderate RI, A	*COL6A3* exon 17 c. 6230 G>A het; (p.Gly2077Asp)^38^
7	22/W	BM	CHD, first steps age 2.5 y, FC, DL, SC, moderate RI, A	*COL6A2* exon 7 c.883 G>A het; (p.Gly295Arg)^38^

*Note*. Moderate respiratory insufficiency corresponds to a FVC between 50% and 70%; severe respiratory insufficiency corresponds to a FVC less than 50%.^7^A, ambulant; CHD, congenital hip dislocation; DL, distal laxity; FC, finger contractures; MV, nocturnal mechanical ventilation; NA, not ambulant; RI, respiratory insufficiency; SC, skin changes (keloid formation, follicular hyperkeratosis).

### Autophagic markers are increased in patients' muscles after one year of LPD

**Figure 1.**
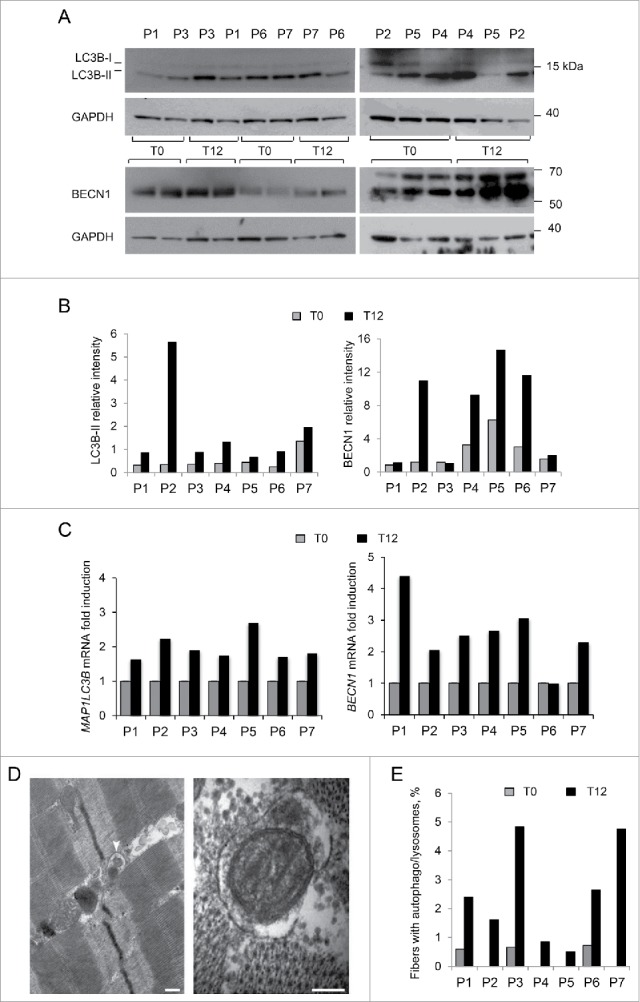
Analysis of autophagy in muscle biopsies of BM and UCMD patients before and after LPD trial. Muscle biopsies from 7 adult patients (P1-P7) were taken before (T0) and after 12 mo LPD (T12). (A) Western blot of MAP1LC3B (LC3B) and BECN1 protein levels in lysates of muscle biopsies at T0 and T12. GAPDH was used as a loading control. Data are representative of at least 3 technical replicates. (B) Densitometric quantification of western blots for LC3B and BECN1. LC3B-II and BECN1 protein levels were calculated as relative intensity with respect to GAPDH. Data represent the mean values (3 experiments) for each patient at T0 and T12. *P* = 0.018 and *P* = 0.028, respectively, for the comparison of LC3B-II and BECN1 mean values between T0 and T12 (Wilcoxon signed rank test for paired data). (C) Quantitative RT-PCR analysis of *MAP1LC3B* and *BECN1* mRNA levels at T0 and T12. Data represent the mean value (2 experiments) for each patient at T0 and T12. *P* = 0.018 and *P* = 0.028, respectively, for the comparison of *MAP1LC3B* and *BECN1* mean fold induction between T0 and T12 (Wilcoxon signed rank test for paired data). (D) Transmission electron micrographs of muscle biopsies after one-y LPD. Representative images of autophagosomes detected in muscle fibers of patient 1 (left panel) and patient 3 (right panel) containing degraded material (left panel, white arrowhead) and a degenerating mitochondrion (right panel). Scale bars: 200 nm. (E) Quantification of myofibers with autophagosomes/autolysosomes, as determined by transmission electron microscopy analysis of muscle biopsies from patients P1-P7 at T0 and T12. The graphs show the percentage of myofibers containing autophagosomes or autolysosomes from 5 different levels for each biopsy. *P* = 0.016, for the comparison between T0 and T12, following Wilcoxon signed rank test for paired data.

**Figure 2.**
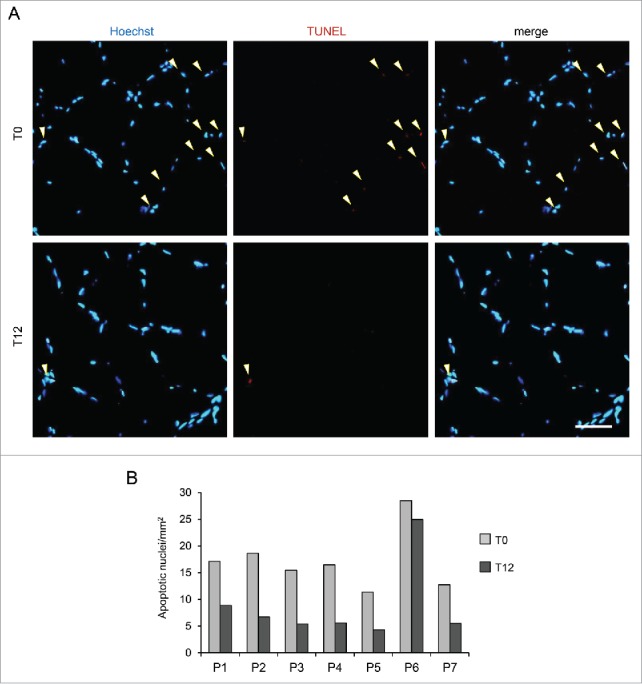
TUNEL assay on muscle biopsies of BM and UCMD patients before and after LPD trial. Muscle biopsies from the 7 patients (P1-P7) were analyzed for apoptosis incidence before (T0) and after a 12-mo LPD (T12). (A) Representative micrographs of TUNEL assay on muscle biopsies of patient 2 at T0 (upper panels) and T12 (lower panels). Nuclei were stained in blue with Hoechst, TUNEL-positive nuclei are in red. Scale bar: 50 µm. (B) Quantification of TUNEL-positive nuclei per area in muscle biopsies from patients P1-P7 at T0 and T12. Mean values from 2 technical replicates of TUNEL assay experiments per condition are shown. *P* = 0.016, following Wilcoxon signed rank test for paired data, for the comparison between mean values at T0 and T12.

The autophagy status was assessed in muscle biopsies by monitoring LC3B lipidation. We also monitored the protein levels of BECN1, a key component of the autophagic machinery that is reduced in *col6a1* null animals and in UCMD and BM patients.^15,16^ When we assessed the levels of lipidated LC3B (LC3B-II), we found a positive effect of the diet on autophagy (Fig. 1A and B). Indeed, the majority of the patients showed increased LC3B-II levels after a 12-mo LPD (T12). Interestingly, patient 5 showed a reduction in total LC3B protein content (both LC3B-I and -II) after the LPD (Fig. 1A), although the corresponding transcript was induced with respect to the baseline condition (T0) (Fig. 1C), suggesting the possibility that in this patient LC3B protein was degraded by sustained autophagy flux. In addition, 4 out of 7 patients had an increase of BECN1 protein levels after the diet (Fig. 1A and B). Notably, the mean values of these autophagic markers were significantly increased in the patient cohort after one y of LPD (*P* = 0.018 for LC3B-II, *P* = 0.028 for BECN1). The expression of *MAP1LC3B* and *BECN1* genes was also increased with respect to the baseline visit, as shown by the increase in mRNA levels (Fig. 1C; *P* = 0.018 for *MAP1LC3B, P* = 0.028 for *BECN1*, for the comparison between T0 and T12). Moreover, electron microscopy of muscle biopsies from patients 1 and 3 at T12 revealed the presence of early autophagosomes containing mitochondria within several myofibers (Fig. 1D), and quantitative analysis showed an increased number of myofibers with autophagosomes and/or autolysosomes in all patients at T12, with respect to T0 (Fig. 1E; *P* = 0.016 following Wilcoxon test for paired data). Western blot analysis for PARK2/parkin and mitochondrial proteins, such as COX4I1/COX IV and TOMM20, did not show any major difference in patients' muscle biopsies between T0 and T12 (Fig. S1). In addition, as previously reported for *col6a1* null animals,^15,20^ reactivation of autophagy promoted myofiber survival. Indeed, the incidence of apoptotic nuclei, as assessed by TUNEL assay in muscle biopsies, was decreased in 6 of the 7 patients after one y of LPD (Fig. 2A and B), and the mean value of apoptotic nuclei was significantly decreased at T12 (*P* = 0.016). Altogether, these results indicate that one-y LPD activated autophagy in muscle biopsies of patients affected by COL6-related myopathies.

### Body composition and clinical parameters remain stable or are mildly improved after one year of LPD

**Figure 3.**
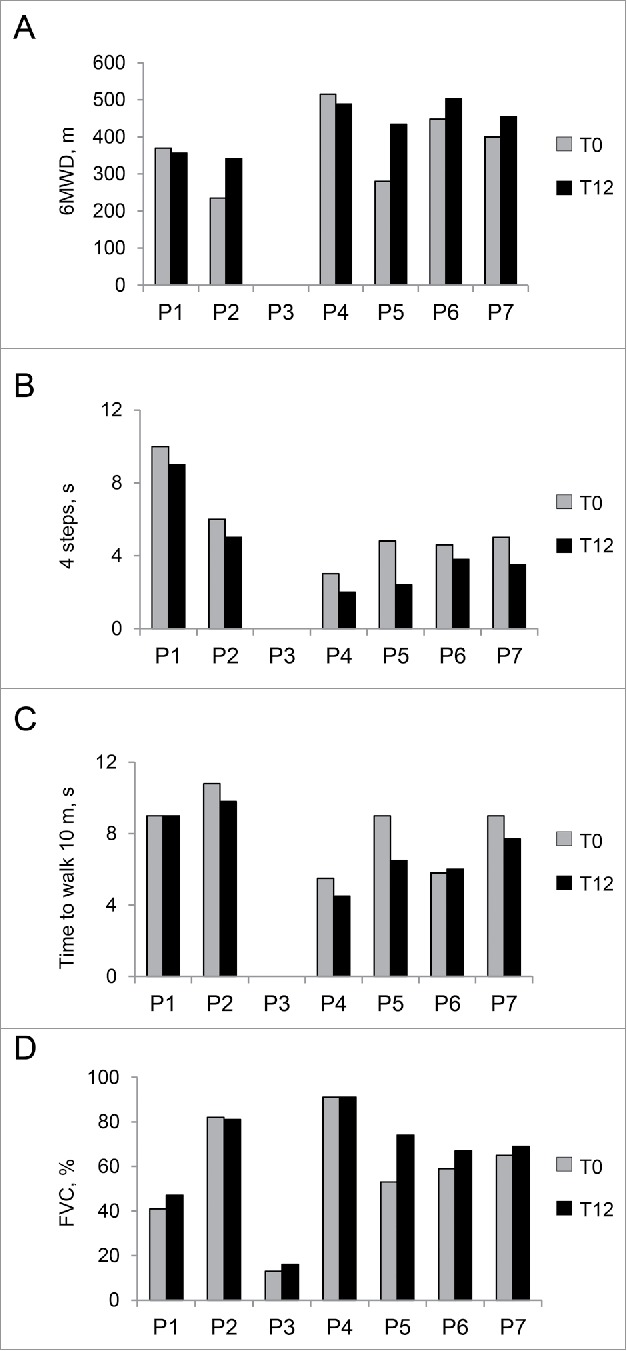
Clinical parameters of the patients before and after LPD trial. (A-C) Motor function parameters in each of the 6 patients able to walk at T0 and T12. (A) Distance walked in 6 min (6MWD), measured in meters (m). (B) Timed 4-stair climb test (4 steps), measured in seconds (s). *P* = 0.034, following Wilcoxon signed rank test for paired data, for the comparison between mean values at T0 and T12. (C) Timed 10-min walk test (10 m), measured in seconds (s). (D) Forced vital capacity percentage (FVC, %) in each of the 7 patients. The comparison between the average FVC percentage at T0 and T12 shows a trend toward increase close to significance (*P* = 0.059) at T12, with respect to baseline visit (T0).

As determined by dietary records, the mean protein intake before the LPD was 1.3 g/kg/day (range 0.8-2.1), and during the trial was 0.63 g/kg/day (range 0.60-0.68). Body mass index (BMI) at T0 showed that one patient was slightly below normal weight (patient 4), and 2 patients were overweight (patients 5 and 7). However, the dual energy X-ray absorptiometry (DXA)-based percentage of fat mass and appendicular lean mass (ALM) index showed that all patients except one (patient 4) were obese, and all except one (patient 7) were also sarcopenic (Table S2). During the trial, patients lost 9% body weight (4.17 ± 2.01 kg), 7% lean mass (2.26 ± 2.45 kg), and 8% fat mass (1.91 ± 3.15 kg). However, there was no statistically significant difference in the fat and lean mass percentage before and after one year of LPD (Table 2). After treatment, ALM decreased less than 0.6 kg in 3 patients and increased in 2 (0.2 and 1 kg); the 2 overweight patients lost 3 and 4 kg (Table S2). Biochemical blood parameters did not show any sign of protein-calorie malnutrition. Notably, despite the minimal decrease in lean tissue mass, there was no significant worsening of any muscle strength parameter including hand grip, elbow flexion, knee flexion, and knee extension (Tables 2 and S2). More importantly, motor function was improved as shown by the improvements in the functional parameters. Mean values of the distance walked in 6 min (6MWD) and of the timed 10-m test (10 m) did not show significant changes and did not worsen as expected, whereas the time to climb 4 steps (4 steps) improved significantly (Table 2**;** Fig. 3A-C). The beneficial effect of the LPD was also displayed by respiratory muscles, with improved respiratory function and an increase in forced vital capacity (FVC) percentage very close to significance (Table 2; Fig. 3D).

**Table 2.** Average values of clinical parameters for all patients before (T0) and after (T12) the LPD trial.

	T0	T12	*P* value*
DXA total mass (kg)	62.07	57.90	0.047
BMI (total mass/H^2^)	22.64	21.15	0.047
FM (%)	44.36	44.51	1.000
FFM (%)	55.64	55.49	1.000
ALM index (kg/m^2^)	4.55	4.16	0.219
HG mean R & L (kg)	15.14	13.84	0.297
EF mean R & L (kg)	5.43	5.91	0.813
KE mean R & L (kg)	10.69	10.84	0.937
KF mean R & L (kg)	8.86	9.00	0.938
6MWD (m)^§^	321	368	0.142
FVC (mL)	2557	2744	0.375
FVC (%)	57	63	0.059
10 m (s)^§^	8.18	7.25	0.104
4 steps (s)^§^	5.57	4.28	0.034

*Note*. *Wilcoxon signed rank test for paired data.

^§^6MWD, 10 m, and 4 steps data are referring to the 6 BM patients able to walk.

EF, elbow flexion; HG, hand grip; FFM, fat-free mass; FM, fat mass; KE, knee extension; KF, knee flexion; R & L, right and left limbs.

### Mitochondria-related metabolic parameters are enhanced after one year of LPD

**Figure 4.**
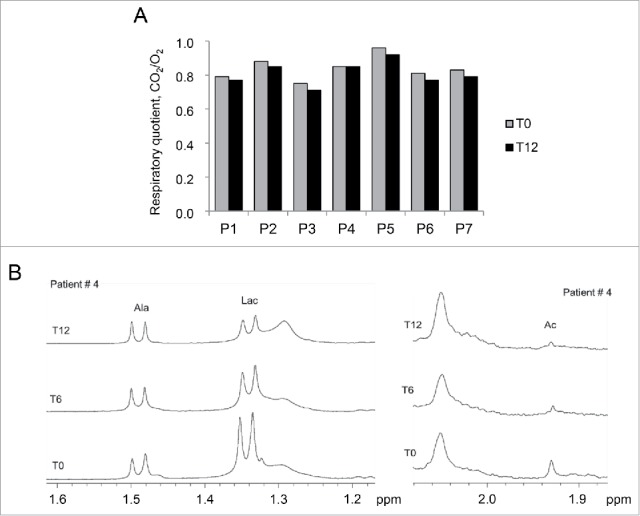
Metabolic parameters of the patients before and after the LPD trial. (A) Histogram showing the respiratory quotient (RQ) calculated at T0 and T12 for each patient (P1-P7). RQ values of each patient at T0 and T12 are listed in Table S3. *P* = 0.031 for the comparison between T0 and T12, following Wilcoxon signed ranked test. (B) Representative ^1^H Carr-Purcell-Meiboom-Gill NMR spectra of serum of patient 4 at T0, T6 and T12, showing the methyl resonance of alanine (Ala), lactate (Lac) and acetate (Ac). Lac:Ala ratios in sera of all patients at T0, T6 and T12 are reported in Table 3.

To evaluate whether activation of autophagy affected mitochondrial function and respiration, we monitored basal metabolism and mitochondria-related metabolites in patients before and after the diet. Notably, the respiratory quotient (RQ) value was reduced in all patients at T12, with statistical significance for the comparison with the RQ at T0 (*P* = 0.031), pointing at enhanced lipid oxidation (Fig. 4A; Table S3). Since β-oxidation of free fatty acids is a mitochondrial process,^21^ this finding indicates that LPD induced a metabolic shift that is consistent with an enhanced mitochondrial oxidative activity. In agreement with this, all patients displayed a reduction in lactate (ranging from 10% to 60%) and a decrease in acetate levels at T12, as revealed by nuclear magnetic resonance (NMR) analysis (Table 3; Fig. 4B). The reduced concentration of lactate and acetate after LPD suggests an improvement in mitochondrial aerobic energy production through the tricarboxylic acid cycle, with a reduced conversion of pyruvate to lactate.

**Table 3.** Lactate:alanine ratios in sera of all patients at T0, T6 and T12, and percentages of lactate reduction (Δ%) after the LPD.

Patient		P1	P2	P3	P4	P5	P6	P7
Lac:Ala ratio*	T0	10: 1	2: 1	3: 1	3: 1	4: 1	2.8: 1	2.7: 1
	T6	3.2: 1	3.2: 1	2.6: 1	2.2: 1	3.7: 1	1.9: 1	2.6: 1
	T12	3.4: 1	1.9: 1	1.4: 1	1.8: 1	3.9: 1	1.7: 1	2.1: 1
Δ%^§^		66	10	53	40	10	40	22

*Note*. *Alanine amount was considered constant in all samples and confirmed by comparison with an external sample of known concentration (set as 8.6 × 10^−5^M).

^§^Δ% is defined as (C_T0_-C_T12_/C_T0_)x100, where C is the alanine concentration at time T0 and T12.

### Blood leukocytes mirror muscle autophagy

**Figure 5.**
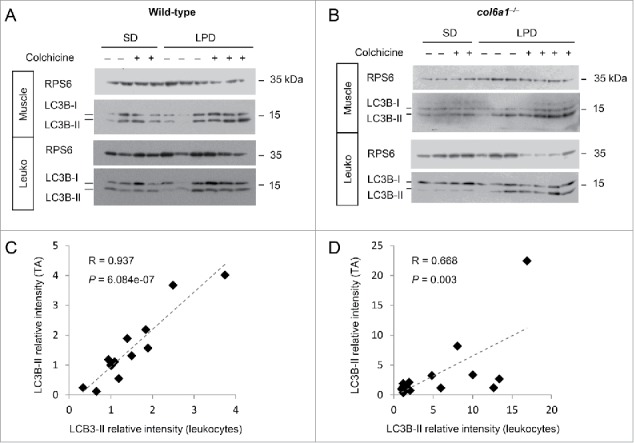
Autophagy activation after LPD is similar in muscles and in leukocytes of wild-type mice and *col6a1* null mice. (A, B) Western blotting of MAP1LC3B/LC3B in protein lysates of *tibialis anterior* muscles and blood leukocytes (Leuko) from wild-type and *col6a1* null (*col6a1*^*−/−*^) mice fed either with standard diet (SD) or low-protein diet (LPD) for 30 d. Each lane corresponds to one animal. For each condition, animals were injected with 0.4 mg/kg/d of colchicine at the end of the diet, to visualize the autophagic flux. RPS6 was used as a loading control. (C, D) Scatter plots showing linear association (dotted line) between LC3B-II relative intensity in *tibialis anterior* (TA) and leukocytes from western blot analysis as shown in (A and B). Relative protein levels of LC3B-II in muscles and leukocytes were quantified vs RPS6. Each point corresponds to one animal. Pearson coefficients (R) and *P* values are shown.

To identify biomarkers that mirror autophagy in muscle, we assessed autophagy in peripheral leukocytes isolated from blood samples. We first used the *col6a1* null mouse model of COL6 deficiency to assess whether LPD triggers autophagy in both muscle and leukocytes. Wild-type and *col6a1* null mice were fed by either standard diet or LPD for 30 d, as previously described,^15^ and LC3B lipidation was evaluated in skeletal muscle and leukocytes (Fig. 5; Fig. S2). Moreover, the autophagic flux was monitored by treating animals with colchicine to block autophagosome-lysosome fusion.^22^ Quantification of western blots showed a significant activation of autophagy by LPD in both muscles and leukocytes (Fig. 5A and B). Importantly, LC3B lipidation in blood leukocytes correlated with skeletal muscle, as revealed by linear regression analysis. LC3B-II levels showed a significant linear correlation between muscle and leukocytes both for wild-type and *col6* knockout mice (Pearson correlation, *P* = 6.084e-07 for wild-type mice; *P* = 0.003 for *col6a1* null mice) (Fig. 5C and D). Altogether, these data revealed that, in rodents, leukocytes mirror the autophagic flux of wild-type and COL6-deficient muscles and therefore may be used as biomarkers.

**Figure 6.**
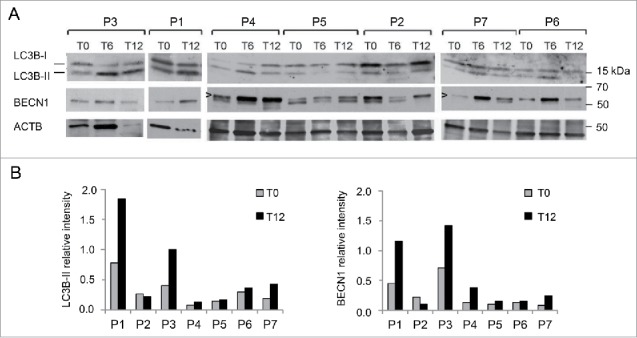
Analysis of autophagy in blood leukocytes of BM and UCMD patients before and after the LPD trial. (A) Western blot of MAP1LC3B/LC3B and BECN1 levels in protein lysates of blood leukocytes at T0, T6 and T12. For patient 1, blood sample at T6 was not available. ACTB was used as a loading control. Arrowheads point at the specific band of BECN1. Data are representative of 2 technical replicates. (B) Densitometric quantification of western blot for LC3B and BECN1 in leukocyte fractions at T0 and T12. LC3B-II and BECN1 protein levels were calculated as relative intensity with respect to ACTB. Data represent mean value at T0 and T12 for each patient (2 experiments). *P* = 0.042 and *P* = 0.062, respectively for the comparison of LC3B-II and BECN1 values between T0 and T12 (Wilcoxon signed rank test for paired data).

To translate these findings to humans, we monitored autophagy in patients' leukocytes before (T0), during (T6) and after (T12) the LPD trial. In agreement with the data obtained in muscle biopsies, leukocytes of most patients showed an increase of LC3B lipidation and of BECN1 levels after LPD (Fig. 6A and B; Fig. S3A; *P* = 0.042 for LC3B-II, *P* = 0.062 for BECN1, for the comparison between T0 and T12 in the patient cohort). Interestingly, western blot analysis showed a peak of LC3B lipidation at T6 in patients 6 and 7, and an early increase in LC3B lipidation at T6 in patient 4, suggesting a time-dependent adaptation to the diet (Fig. S3B). Similar to muscle, leukocytes of patient 5 displayed minor changes of LC3B lipidation. Conversely, leukocytes of patient 2 differed from muscle and showed small changes in the LC3B-II band (Fig. 6A and B). Correlation analyses for the western blot data obtained before and after LPD showed that the BECN1 levels of muscle biopsies and leukocytes faintly correlated at T0 (R = 0.467, *P* = 0.091) and significantly correlated at T12 (R = 0.630, *P* = 0.033), whereas no correlation was found between muscles and leukocytes for the LC3B-II protein levels (baseline visit: R = 0.085, *P* = 0.527; T12 visit: R = 0.139, *P* = 0.410). Importantly, the mean values of these markers in leukocytes at T0 and T12 showed that LC3B lipidation was significantly increased after one y of LPD (*P* = 0.042), whereas BECN1 levels displayed a trend toward increase, near to statistical significance (*P* = 0.062). Altogether, these results indicate that, similar to muscle biopsies, autophagy activation occurred in patients' leukocytes after one y of LPD.

## Discussion

The findings of this nutritional pilot trial, although carried out on a small cohort of patients affected by a rare disease, provide valuable information for the medical community. Indeed, this is the first clinical study showing autophagy activation in an inherited human disorder, with beneficial effects in counteracting disease progression. The improvement of multiple functional parameters, despite some degree of body weight loss, is a notable outcome, confirming the safety and the potential benefits of activating autophagy in COL6-related myopathies. Our data suggest that LPD in human COL6 myopathies recapitulates the beneficial effects seen in *col6a1* null mice.^15^ Similar to the animal model, this nutritional approach was capable of inducing autophagy, a pathway playing a critical role in the removal of damaged organelles and dysfunctional mitochondria, leading to improved myofiber health.^23^

Given the early onset and progressive course of COL6-related myopathies, patients typically get worse with time, but not in a predictable way.^1^ LPD treatment is expected to enhance autophagy-mediated protein and organelle breakdown and, as a consequence, patients lost 7% of muscle mass. However, our data indicate that despite a certain degree of muscle mass loss and the fact that patients aged, the absolute force of leg muscles did not decrease after one-y LPD, and activation of autophagy resulted in decreased myofiber apoptosis, better motor function and improved respiration. These apparently paradoxical findings are readily explained by the critical control that autophagy elicits on the quality of proteins and organelles.^16,17,24^ By promoting the autophagic flux, damaged organelles and proteins are cleared, and muscles lose some of their mass but become better in function. Conversely, autophagy inhibition in muscle is sufficient to induce wasting and weakness.^25,26^ Consistent with our data, a recent work showed that long-term calorie restriction in healthy human volunteers by increasing BECN1 and LC3B levels promotes beneficial changes in skeletal muscles, such as reduced inflammation and better proteostasis.^27^

It can be argued that the metabolic shift toward lipid oxidation we detected in all patients after one y of LPD relies upon the improved mitochondrial function elicited by autophagy activation. This is consistent with the fact that fatty acid β-oxidation is one of the main energy metabolism pathways operating inside mitochondria. The observation that serum lactate and acetate decreased following one y of LPD further points at a more efficient mitochondrial oxidative activity. We underline that our data represent a “static” analysis of autophagy and cannot provide insights about the autophagic flux, due to the lack of appropriate methods for flux detection in humans.^18^ This implies that autophagy activation may be present even in those patients displaying small or no changes in LC3B lipidation and/or BECN1 levels.

The limitations of this pilot clinical study are unavoidably linked to the low prevalence of BM and UCMD^1,5,8^ and to the small number of patients eligible for such a pilot trial. Indeed, it is extremely difficult to design randomized trials for patients affected by COL6-related myopathies or to include a standard diet control group for the LPD, due to obvious ethical constraints raised by the invasive multiple muscle biopsies required for evaluating the efficacy. In spite of these intrinsic limitations, our findings are very promising and pave the way for nutritional interventions aimed at autophagy activation in neuromuscular pathologies. Actually, the goal of this trial was investigating the usefulness of targeting autophagy in COL6-related myopathies, thus providing the foundation and justifying future larger clinical studies. It should be underlined that this pilot clinical trial is not aimed at recommending LPD as a definitive long-term treatment for BM or UCMD patients. Instead, our study was finalized at assessing whether autophagy can be activated in BM and UCMD patients, and whether such activation is safe and has beneficial effects toward a future therapy. Moreover, by using nutritional intervention as a simple and safe approach to activate autophagy and by monitoring vital signs of patients, we were able at this initial step to exclude from the analysis additional side effects that could have been attributed to a drug or a chemical autophagy activator.^24^

There is an increasing need to identify biomarkers and tools for monitoring autophagy in humans without using biopsies.^18,24^ Our work is the first study demonstrating that blood leukocytes represent a promising noninvasive tool for assessing autophagy status in neuromuscular diseases, both in animals and in humans. This finding is particularly relevant for tissues not easily accessible to biopsy, such as brain and heart, and in which autophagy is an important homeostatic player. In fact, autophagy is a valuable and druggable target for combating neurodegenerative disorders, such as Huntington, Alzheimer and Parkinson diseases, as well as congenital or acquired cardiomyopathies.^16,17,28,29^ Finally, our data also support the possibility to study the autophagy status in humans by collecting and using blood leukocytes for biochemical measurements.

Altogether, our data successfully fulfilled the goals of this pilot LPD clinical trial, demonstrating not only that autophagy can be activated in myopathic patients, but also that autophagy is a valuable target for novel therapeutic approaches. In the near future, the combination of natural compounds that modulate autophagy^20^ with specifically designed nutritional programs may allow synergizing their actions and further improving the mild benefits obtained in this trial by the LPD. In conclusion, the results we obtained by one-y LPD treatment in BM and UCMD patients open the field for further studies in other inherited myopathies and muscular dystrophies in which autophagy impairment is part of the pathogenic mechanisms causing muscle wasting and weakness.^23^

## Patients and methods

### Study design and objectives

This was a 12-mo, open-label, noncomparative, single-arm, pilot study on the efficacy, safety, and tolerability of LPD in adult patients with the diagnosis of BM or UCMD. Patients were recruited between November 2011 and February 2012, and a one-y LPD was carried out from March 2012 until June 2013. The study project was scientifically supervised by Luciano Merlini, MD, and the clinical study was located at the Rizzoli Orthopedic Institute, Bologna, Italy. The study was performed in accordance with the Declaration of Helsinki and was approved by the Medical Ethics Committee of the Rizzoli Orthopedic Institute. This study is registered with ClinicalTrials.gov, number NCT01438788. The primary endpoint of the trial was activation of autophagy measured as a change in biological markers of autophagy in muscle biopsy from baseline to day 365. The secondary outcome measures included safety and changes in nutritional parameters, muscle mass, muscle strength and function. A detailed study flow is shown in Table S1.

### Enrollment criteria

Patients aged 18 y or older, with the clinical^30^ and molecular diagnosis of UCMD or BM, and with no serious internal medicine complications were eligible. Patient recruitment was subordinated to the adherence to the following criteria: women of childbearing age must have a negative pregnancy test and must use adequate contraception during the study; no previous treatment with cyclosporin A within 6 mo prior to the start of the study; willingness and ability to adhere to the study visit schedule and other protocol requirements; ability to provide written informed consent. Pregnant or breast-feeding women, and patients with current or history of liver or renal disease, or with any serious internal medicine condition interfering with the study were not eligible.

### Patient evaluation

Safety evaluation in blood and urine, general and nutritional status, and adherence to the diet were assessed every month from the baseline visit (see Supplemental Material for further details). Biopsies of the tibialis anterior muscle were obtained at the beginning (T0) and at the end (T12) of the trial. In addition, BMI, circumferences (waist, hip), and body composition by DXA, were also assessed at baseline (visit T0), 6 mo (visit T6), and 12 mo (visit T12). Muscle strength and motor function were assessed at T0 and T12; leukocytes were collected at T0, T6, and T12.

### Nutritional status and diet

Body composition determined by DXA (Hologic 4500 W; software version 11.2; Hologic Inc., Waltham, MA) included estimation of whole body mass, ALM, fat mass, and bone mineral content (Table S2). Sarcopenic obesity was defined as ALM divided by stature squared < 7.26 kg/m^2^ in men and 5.45 kg/m^2^ in women, and as percent body fat greater than 28% in men and 40% in women.^31^ Anthropometric measurements included body weight, height, BMI and circumferences (waist, hip). Resting energy expenditure and RQ were assessed in the post-absorptive state by indirect calorimetry (CareFusion Vmax Encore, San Diego, CA). Energy and macronutrient intake were evaluated with a 7-d dietary record. The Metadieta software (Meteda S.r.l., San Benedetto del Tronto, Italy) was used to calculate energy, protein, carbohydrate and lipid contents of the diet. Food preferences or intolerances were assessed via a dietitian interview. Lifestyle was evaluated during the interview. The nutritional intervention consisted of a personalized LPD with a quantity of calories that matched energy expenditure.^32^ Energy requirement was calculated as the product of resting energy expenditure (kcal/day) and physical activity level. Daily protein content of the LPD was up to 0.68 g protein/kg body weight. The amounts of carbohydrates and lipids in the diet were calculated as a percentage of the total daily energy requirements after withdrawal of calories derived from proteins (Table S4). In the case of overweight patients (25.0 < BMI < 29.9), the protein amount was calculated on the basis of height and an ideal body weight corresponding to a BMI of 22.5 kg/m^2^. The reduced quantity of proteins in the diet was obtained through low-protein products such as pasta, bread, or biscuits (Aproten, Latina, Italy) provided monthly to the patients. Empty packets were collected to determine compliance. High quality animal proteins were kept in the diet. The weekly menu was planned with consideration of the patient's food preferences. A dietitian was available by daily phone contact in addition to the monthly meetings to ensure long-term adherence to the diet.

### Safety assessment

At baseline and then every month, all patients underwent a full physical examination with measurements of vital signs. Plasma and urine were obtained to determine liver and renal function, electrolyte levels, and cell counts (see Supplemental Material). The use of concomitant medications and the number of adverse events were recorded.

### Muscle strength and function

Maximal isometric strength was assessed using a hand-held dynamometer (Type CT 3001, Citec, C.I.T. Technics BV, Groningen, The Netherlands).^33^ Four muscle movements were examined bilaterally: hand grip, elbow flexion, knee flexion and knee extension.^34-36^ The maximal force from 3 attempts was used for data analysis. FVC was determined with an electronic spirometer, and percent-predicted values were calculated based on published normal values.^7^ The timed 10-min walk test (10 m), 4-stair climb test (4 steps), and the distance walked in 6 min (6MWD) were also assessed.

### Muscle biopsy and electron microscopy

Part of the biopsy was frozen in liquid nitrogen and used for western blot and TUNEL test, while the remaining portion was fixed with 2.5% glutaraldehyde in 0.1 M cacodylate buffer and processed for electron microscopy as previously described.^37^ For quantitative analysis of myofibers with autophagosomes or autolysosomes, we analyzed at least 200 muscle fibers obtained from 5 different levels for each biopsy.

### Blood sample collection

Blood samples at baseline, visit T6, and visit T12 were always collected at the same time in the morning, in starved patients (pre-prandial condition). At T0 and T12, just after blood collection, each patient underwent muscle biopsy to be analyzed in correlation with a blood sample.

### RNA extraction and quantitative RT-PCR

Sections (20-µm thick) were cut from muscle biopsies and RNA extraction was performed using TRIzol reagent (Life Technologies, 15596-018) according to manufacturer's instructions. Total RNA (500 ng) were retrotranscribed with the SuperScript III kit (Life Technologies, 18080-051) following the manufacturer's instructions. Quantitative PCR was performed on a RotorGeneQ instrument (Qiagen), using specific primers and a master mix containing CyBr green (Qiagen, 204076). Primer sequences are provided in Table S4. RNA analyses were performed as single-blind tests.

### *In situ* TUNEL assay

TUNEL assay was performed with the In situ Cell Death Detection Kit, TMR red (Roche, 12156792910), on 7-μm-thick muscle sections prepared from the cryopreserved biopsies. After 20 min of fixation (4% paraformaldehyde in phosphate-buffered saline, pH 7.4, Santa Cruz Biotechnology, sc-281692), slides were washed in phosphate-buffered saline and permeabilized for 2 min in 0.1% Triton X-100 (Sigma, T9284), 0.1% sodium citrate. Enzymatic reaction was performed according to the manufacturer's guidelines. Nuclear counterstaining was obtained using Hoechst 33258 (Sigma-Aldrich, B1155). The number of TUNEL-positive nuclei was calculated in at least 25 randomly selected fields per biopsy by using a Zeiss Axioplan microscope equipped with a digital camera, and then was compared with the relative sampled area. Mean values are representative of 2 technical replicates of TUNEL experiments for each condition.

### NMR measurement

Serum samples were collected from patients at T0, T6 and T12 and prepared directly into 5-mm NMR tubes. Frozen samples were thawed at room temperature and 400 μl were supplemented with 100 μl D_2_O. NMR spectra were acquired at 400.13 MHz on a spectrometer (Avance400, Bruker) at 298 K. For each sample one-dimensional ^1^H NMR was acquired using a water-suppressed spin-echo Carr-Purcell-Meiboom-Gill implemented sequence (Bruker Software, MA, USA). The spectra were referenced to the alanine doublet at 1.49 ppm.

### Animals

C57BL/6N mice were obtained from Charles River. *col6a1* null (*col6a1*^−/−^) mice were generated in our laboratory as previously described.^10,20^ All mice were housed in a controlled environment with 12-h light/12-h dark cycle and a temperature of 23°C. Mice were provided with *ad libitum* standard chow and water, and experiments were carried out at 9 a.m. on sex- and age-matched mice with free access to the water, as previously described.^20^ For the LPD, mice were fed *ad libitum* for 1 mo with either standard chow (Laboratorio Dottori Piccioni, #52) or 5% protein purified chow (TestDiet, T-5767-7155) with free access to water, as previously described.^15^ During the last 2 d of standard diet or LPD, mice received daily intraperitoneal injections with 0.4 mg/kg/day of colchicine (Sigma-Aldrich, C9754), an inhibitor of autophagosome-lysosome fusion, allowing the investigation of the autophagic flux.^22^ All the animals were sacrificed by cervical dislocation, blood was collected by cardiac puncture and the tissues of interest were dissected and fixed. Peripheral mononuclear blood cells were separated by density centrifugation (Ficoll-Paque PREMIUM 1.084, 17-5446-02, GE Healthcare) and lysed in RIPA buffer (20 mM Tris-HCl, pH 7.0, 1% Nonidet P-40 [Sigma, I8896], 150 mM NaCl, 10% glycerol, 10 mM EDTA, 20 mM sodium fluoride, 5 mM sodium pyrophosphate, 1 mM Na_3_VO_4_, 1 mM phenylmethylsulfonyl fluoride), supplemented with phosphatase and protease inhibitors (Sigma, P5726). At least 3 animals per experimental condition were analyzed.

### Western blotting

Frozen muscle biopsies were pulverized and lysed as previously described.^15^ Human peripheral blood leucocytes were separated by density centrifugation (LMS 1077, PAA Laboratories) and lysed in RIPA buffer containing protease inhibitors. For each sample, an equal amount of lysate (30 µg for human muscle lysates, 50 µg for mouse muscle lysates, 20 µg for human and mouse leukocytes) was reduced by exposure to 20% β-mercaptoethanol and subjected to SDS-PAGE as previously described.^15^ Proteins were transferred overnight onto a PVDF transfer membrane (Thermo Scientific, 88518) at 4°C. After blocking with 5% dry milk solution, membranes were probed with the following primary antibodies: anti-BECN1 (1:1000; Cell Signaling Technology, 3738), anti-LC3B (1:800; Novus, NB-100 2220; and 1:1000; Sigma-Aldrich, L7543); anti-ACTB/β-ACTIN (1:1000; Santa Cruz Biotechnology, sc-1616); anti-GAPDH (1:50,000; Millipore, MAB374); anti-RPS6/ribosomal protein S6 (1:1000; Cell Signaling Technology, 2217); anti-PARK2 (1:500; Santa Cruz Biotechnology, sc-32282); anti-COX4I1 (1:2000; Cell Signaling Technology, 4844); anti-TOMM20 (1:250; Santa Cruz Biotechnology, sc-11415). Biochemical analyses on human muscle samples were performed as single-blind tests. Suitable loading controls were used for each tissue (GAPDH for muscle protein lysates, RPS6 for mouse leukocyte lysates). Red Ponceau staining of total protein content was also performed for all western blotting experiments (not shown).

### Study approval

All participants were provided written informed consent, and approval was obtained from the Ethics Committee of the Rizzoli Orthopedic Institute (ClinicalTrials.gov, number NCT01438788). Animal experiments were carried out according to all pertinent Italian laws and were approved by the Ethics Committee of the University of Padova.

### Statistics

We used a nonparametric 2-tailed Wilcoxon signed-rank test for paired samples to determine whether there was a significant difference in all the variables from T0 to T12. Pearson correlation analysis was used to determine the relationship between autophagy induction in muscles and blood leukocytes, and the correlation coefficient (R) and *P* value are given. *P* values < 0.05 were considered statistically significant. Mean values obtained from technical replicates (western blotting, qRT-PCR, TUNEL) on the same biopsy are shown without standard error of the mean, because they belong to the same biological sample. All the aggregated values are reported as means ± standard error of the mean, unless otherwise stated.

## Supplementary Material

Supplementary files

## Abbreviations

ALM: appendicular lean mass
BM: Bethlem myopathy
BMI: body mass index
DXA: dual energy X-ray absorptiometry
FVC: forced vital capacity
LPD: low-protein diet
NMR: nuclear magnetic resonance
RQ: respiratory quotient
UCMD: Ullrich congenital muscular dystrophy
10 m: timed 10-m test
4 steps: test measuring the time to climb 4 steps
6MWD: test measuring the distance walked in 6 min
