# Supplementary material for: Autophagy activation in COL6 myopathic patients by a low-protein-diet pilot trial
Source: Autophagy. 2016 Sep 22;12(12):2484–95. doi: 10.1080/15548627.2016.1231279 (PMC5173266; doi:10.1080/15548627.2016.1231279)
Supplement: Supplementary files [file kaup-12-12-1231279-s001.docx]

**Supplemental Material to:**

Autophagy activation in COL6 myopathic patients by a low-protein-diet pilot trial

Silvia Castagnaro, Camilla Pellegrini, Massimo Pellegrini, Martina Chrisam, Patrizia Sabatelli, Silvia Toni, Paolo Grumati, Claudio Ripamonti, Loredana Pratelli, Nadir M. Maraldi, Daniela Cocchi, Valeria Righi, Cesare Faldini, Marco Sandri, Paolo Bonaldo, and Luciano Merlini

Content:

- Supplementary Methods

- Supplementary Figures 1-3

- Supplementary Tables 1-5**Supplementary Methods**

**Safety evaluation.**

Safety evaluation was performed every month from the baseline visit and included clinical and laboratory tests.

Safety clinical evaluation included:

- physical examination;
- measurements of vital signs (blood pressure, pulse rate, pO_2_, weight, height);
- review of adverse events;
- review of concomitant medications.

Safety laboratory tests included:

- basic metabolic panel (Na, K, Cl, Ca, CO_2_, BUN, creatinine, glucose);
- liver function tests (GOT1/AST, GPT1/ALT, total bilirubin, direct bilirubin, total protein, ALP);
- renal function tests (estimated GFR [eGFR], using modification of diet in renal disease [MDRD] formula);
- complete blood count (WBC, Hct, Hgb, RBC, Plt) and CK/creatine kinase;
- lipids panel, CRP, TSH, IGF-1, albumin, prealbumin and acetone (ketones);
- urinalysis (leukocytes; blood; protein; ketone bodies; bilirubin; urobilinogen; creatinine; glucose; nitrite; pH; urine sediment).

**Diet and nutritional intake tracking.**

The low protein diet was personalized for each patient, taking into account the specific calorie and nutrient requirements (energy requirement was calculated as described in the Methods section), and patients’ food preferences. The resulting personalized diet compositions are shown in Table S4. First, a dietitian taught patients how to follow the diet and they underwent a training period of 4 days, before starting the 1-year trial. Patients recorded their daily food intake using dedicated diaries and were invited for a monthly visit, to ensure long-term adherence to the diet. In addition, a dietitian was available (phone and email contacts) for contingent daily calls, to clarify any doubt or problem with the diet. The first method to check compliance for the diet was the monitoring of the amount of empty packets harvested. Then, at every monthly visit, the adherence to the diet was monitored by a dietitian interview and anthropometric measurements (body weight, height, body mass index and circumferences) were performed. Based on the recording of these parameters, minimal adjustments to the diet were made when needed, to assure the correct nutritional value.


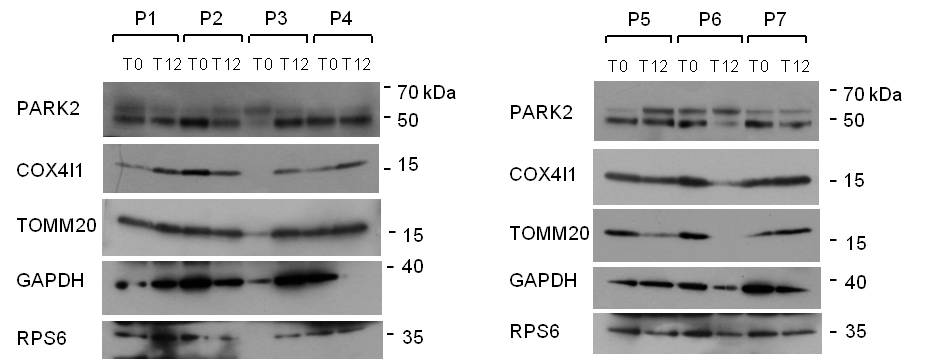
**Figure S1.** Western blot analysis of PARK2/parkin and mitochondrial proteins in muscle biopsies of BM and UCMD patients before and after the LPD trial. Muscle biopsies from patients P1-P7 were taken before (T0) and after 12 months LPD (T12). PARK2, COX4I1/COXIV (cytochrome c oxidase subunit 4I1), and TOMM20 (translocase of outer mitochondrial membrane 20) protein levels in muscle lysates at T0 and T12 are shown. GAPDH and RPS6 were used as a control for total protein content. Data are representative of 2 technical replicates.

**
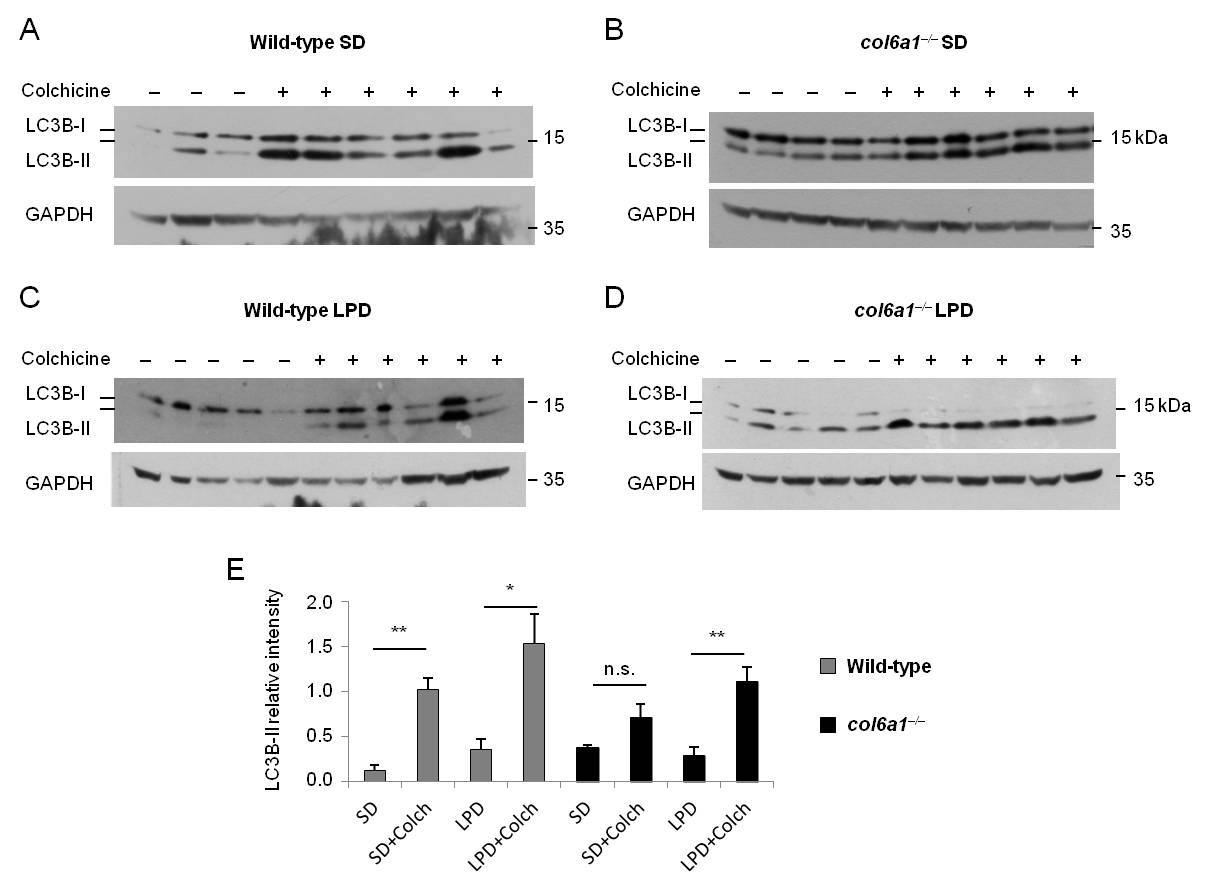
Figure S2.** Western blot analysis of MAP1LC3B/LC3B in protein lysates of *tibialis anterior* muscles of wild-type mice and *col6a1* null mice. Wild-type and *col6a1* null (*col6a1^–/–^*) mice were fed for 30 days either with standard diet (**A, B**) or low-protein diet (**C, D**). Where indicated, animals were injected twice with 0.4 mg/kg/day of colchicine at the end of the diet, to visualize the autophagic flux. GAPDH was used as a control for total protein content. Each lane corresponds to one animal. (**E**) Densitometric quantification of western blots in (**A-D**). LC3B-II protein levels were calculated as relative intensity with respect to GAPDH. Mean values for the same group of animals (SD, SD+Colch, LPD, and LPD+Colch) are shown. (*, *P* < 0.05; **, *P* < 0.01, following two-tailed t test). Colch, colchicine; LPD, low-protein diet; n.s., not significantly different; SD, standard diet.

**
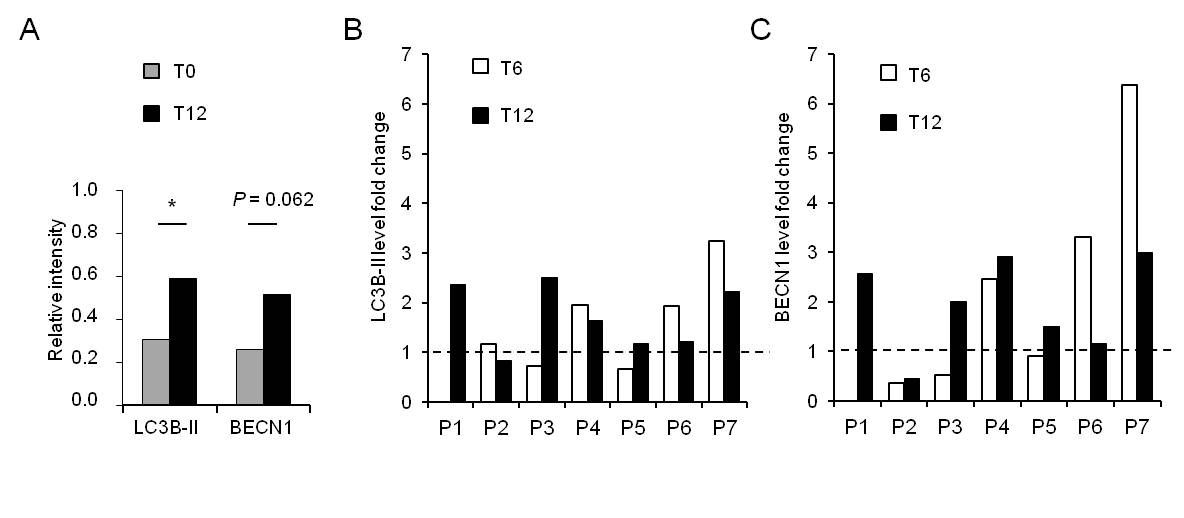
 Figure S3.** Quantification of autophagy proteins in blood leukocytes of BM and UCMD patients. Leukocytes were isolated from patients at day 1 (T0), 6 months (T6) and 12 months (T12) of the LPD trial, and protein lysates analyzed by western blotting as shown in Fig. 6A in the main text. (**A**) Densitometric analysis of western blot for LC3B-II and BECN1. Data represent the mean value for all patients at T0 and T12, recapitulating the data shown in Figure 6B. *, *P* = 0.042, following Wilcoxon signed rank test for paired data. (**B, C**) Densitometric analysis of western blot for LC3B-II (**B**) and BECN1 (**C**) for each patient. LC3B-II and BECN1 protein levels were calculated as relative intensity with respect to ACTB, used as a loading control. The respective protein levels in leukocyte fractions at T6 and T12 were compared to the T0 condition (indicated by the dotted line, arbitrarily set as 1).

**Table S1.** Study flow.

|  |  |  |  |  | **T0** |  |  |  |  | **T6** |  |  |  |  |  | **T12** |  |
| --- | --- | --- | --- | --- | --- | --- | --- | --- | --- | --- | --- | --- | --- | --- | --- | --- | --- |
| Visit | 1 | 2 | 3 |  | 4 | 5 | 6 | 7 | 8 | 9 | 10 | 11 | 12 | 13 | 14 | 15 | 16 |
| Day | ±2 | -84 | -45 | -1 | 1-3 |  |  |  |  | 172 |  |  |  |  |  | 365 | 400 |
| Period | Screening | Observational | | | Treatment | | | | | | | | | | | | Follow-up |
| Eligibility/Informed consent | X |  |  |  |  |  |  |  |  |  |  |  |  |  |  |  |  |
| Medical history | X |  |  |  | X |  |  |  |  |  |  |  |  |  |  |  |  |
| Physical examination | X |  |  |  | X | X | X | X | X | X | X | X | X | X | X | X | X |
| Pregnancy test | X |  |  |  |  |  |  |  |  |  |  |  |  |  |  |  |  |
| Nutritional evaluation |  | X |  |  | X | X | X | X | X | X | X | X | X | X | X | X | X |
| Investigational diet interview | X |  |  |  |  |  |  |  |  |  |  |  |  |  |  |  |  |
| Dietitian training |  | X | X | X |  |  |  |  |  |  |  |  |  |  |  |  |  |
| Anthropometry* |  | X |  |  | X | X | X | X | X | X | X | X | X | X | X | X | X |
| Energy intake^§^ |  | X |  |  | X | X | X | X | X | X | X | X | X | X | X | X | X |
| Indirect calorimetry |  | X |  |  | X |  |  |  |  |  |  |  |  |  |  | X |  |
| Bioimpendance analysis (BIA) |  | X |  |  | X |  |  |  |  | X |  |  |  |  |  | X |  |
| Dual energy X-ray absorptiometry (DXA) |  | X |  |  | X |  |  |  |  | X |  |  |  |  |  | X |  |
| Diet |  |  |  |  | X | X | X | X | X | X | X | X | X | X | X | X |  |
| Empty package collection |  |  |  |  |  | X | X | X | X | X | X | X | X | X | X | X |  |
| Muscle biopsy |  |  |  |  | X |  |  |  |  |  |  |  |  |  |  | X |  |
|  |  |  |  |  | **T0** |  |  |  |  | **T6** |  |  |  |  |  | **T12** |  |
| Visit | 1 | 2 | 3 |  | 4 | 5 | 6 | 7 | 8 | 9 | 10 | 11 | 12 | 13 | 14 | 15 | 16 |
| Day | ±2 | -84 | -45 | -1 | 1-3 |  |  |  |  | 172 |  |  |  |  |  | 365 | 400 |
| Period | Screening | Observational | | | Treatment | | | | | | | | | | | | Follow-up |
| Handheld myometry |  | X |  |  | X |  |  |  |  | X |  |  |  |  |  | X |  |
| Timed tests^¥^ |  | X |  |  | X |  |  |  |  | X |  |  |  |  |  | X |  |
| Spirometry |  | X |  |  | X |  |  |  |  | X |  |  |  |  |  | X |  |
| Urinalysis |  |  |  |  | X | X | X | X | X | X | X | X | X | X | X | X |  |
| Urinanalysis 24-h |  |  |  |  | X |  |  |  |  | X |  |  |  |  |  | X |  |
| Safety biochemistry |  |  |  |  | X | X | X | X | X | X | X | X | X | X | X | X |  |
| Safety hematology |  |  |  |  | X | X | X | X | X | X | X | X | X | X | X | X |  |
| ECG |  |  |  |  | X |  |  |  |  | X |  |  |  |  |  | X |  |
| SF-36 Questionnaire—Quality of life |  |  |  |  | X |  |  |  |  |  |  |  |  |  |  | X |  |
| Adverse events |  |  |  |  | X | X | X | X | X | X | X | X | X | X | X | X |  |
| Vital signs |  |  |  |  | X | X | X | X | X | X | X | X | X | X | X | X |  |
| Concomitant medication |  | X | X |  | X | X | X | X | X | X | X | X | X | X | X | X | X |

*Weight, height, BMI, skinfold thickness and circumferences.

^§^Dietary intake diary.

^¥^10-meter walk/run, rising from floor, stair climb, 6-min walk.

**Table S2.** Clinical parameters for each patient (P1-P7) monitored before (T0) and after (T12) the 1-year LPD.

|  | **Visit** | **T0** | | | | | | | **T12** | | | | | | |
| --- | --- | --- | --- | --- | --- | --- | --- | --- | --- | --- | --- | --- | --- | --- | --- |
|  | **Patient** | **P1** | **P2** | **P3** | **P4** | **P5** | **P6** | **P7** | **P1** | **P2** | **P3** | **P4** | **P5** | **P6** | **P7** |
|  |  |  |  |  |  |  |  |  |  |  |  |  |  |  |  |
|  | DXA total mass (kg) | 58.05 | 68.23 | 56.30 | 54.07 | 77.18 | 46.54 | 74.10 | 57.28 | 63.29 | 55.00 | 55.28 | 62.92 | 45.05 | 66.46 |
|  | BMI (total mass/H^2^) | 21.60 | 23.20 | 22.00 | 18.30 | 25.80 | 18.50 | 29.10 | 21.30 | 21.50 | 21.50 | 18.70 | 21.00 | 17.90 | 26.10 |
|  | FM (%) | 4.10 | 42.50 | 60.80 | 18.90 | 42.40 | 42.30 | 54.50 | 49.30 | 41.20 | 59.80 | 22.80 | 39.70 | 42.20 | 56.60 |
|  | FFM (%) | 50.90 | 57.50 | 39.20 | 81.10 | 57.60 | 57.70 | 45.50 | 50.70 | 58.80 | 40.20 | 77.20 | 60.30 | 57.80 | 43.40 |
|  | ALM index (kg/m^2^) | 3.35 | 4.64 | 2.95 | 5.80 | 5.79 | 3.34 | 5.99 | 3.72 | 4.43 | 3.04 | 5.70 | 4.75 | 3.21 | 4.29 |
|  |  |  |  |  |  |  |  |  |  |  |  |  |  |  |  |
|  | HG mean R & L (kg) | 10.40 | 26.41 | 3.47 | 26.31 | 14.99 | 13.66 | 10.71 | 11.32 | 24.17 | 4.18 | 19.88 | 13.66 | 13.56 | 10.10 |
|  | EF mean R & L (kg) | 4.03 | 7.09 | 1.33 | 10.81 | 6.58 | 3.77 | 4.44 | 3.37 | 11.17 | 1.68 | 9.89 | 7.55 | 3.16 | 4.59 |
|  | KE mean R & L (kg) | 8.11 | 5.10 | 3.31 | 24.32 | 12.75 | 7.04 | 14.23 | 7.55 | 3.72 | 3.77 | 27.12 | 13.31 | 6.63 | 13.77 |
|  | KF mean R & L (kg) | 8.41 | 7.24 | 2.24 | 15.65 | 11.52 | 7.65 | 9.28 | 7.34 | 5.86 | 2.80 | 16.88 | 13.26 | 9.33 | 7.55 |
|  |  |  |  |  |  |  |  |  |  |  |  |  |  |  |  |
|  | 6MWD (m)^*^ | 370 | 235 | *NA* | 515 | 280 | 448 | 400 | 356 | 341 | *NA* | 489 | 434 | 503 | 454 |
|  | FVC (mL) | 1410 | 3990 | 530 | 4730 | 2760 | 1950 | 2530 | 1440 | 3930 | 610 | 4630 | 3820 | 2210 | 2570 |
|  | FVC (%) | 41 | 82 | 13 | 91 | 53 | 59 | 65 | 47 | 81 | 16 | 91 | 74 | 67 | 69 |
|  | 10 m (s)^*^ | 9.00 | 10.80 | *NA* | 5.50 | 9.00 | 5.80 | 9.00 | 9.00 | 9.80 | *NA* | 4.50 | 6.50 | 6.00 | 7.70 |
|  | 4 steps (s)^*^ | 10.00 | 6.00 | *NA* | 3.00 | 4.80 | 4.60 | 5.00 | 9.00 | 5.00 | *NA* | 2.00 | 2.40 | 3.80 | 3.50 |

^*^6MWD, 10 m and 4 steps data are referring to the 6 BM patients that were able to walk. EF, elbow flexion; FFM, fat-free mass; FM, fat mass; HG, hand grip; KE, knee extension; KF, knee flexion; NA, not available; R & L, right and left limbs. The units of measurement are shown in brackets.

**Table S3.** Respiratory quotient values of patients before (T0) and after (T12) the LPD trial.

| **Patient** | **P1** | **P2** | **P3** | **P4** | **P5** | **P6** | **P7** |
| --- | --- | --- | --- | --- | --- | --- | --- |
| T0 | 0.79 | 0.88 | 0.75 | 0.85 | 0.96 | 0.81 | 0.83 |
| T12 | 0.77 | 0.85 | 0.71 | 0.85 | 0.92 | 0.77 | 0.79 |

**Table S4.** Specific LPD composition for each BM or UCMD patient (P1-P7).

| **Patient** | **P1** | **P2** | **P3** | **P4** | **P5** | **P6** | **P7** |
| --- | --- | --- | --- | --- | --- | --- | --- |
| **Energy (kcal/day)** | 1692 | 2160 | 1400 | 2172 | 2412 | 2171 | 1674 |
| **Protein supply (g/kg)**^*^ | 0.65 | 0.60 | 0.62 | 0.61 | 0.62 | 0.65 | 0.68 |
| **Protein (%)** | 8.58 | 7.67 | 9.96 | 6.26 | 7.03 | 5.52 | 8.98 |
| **Carbohydrate (%)**^§^ | 61.12 | 61.24 | 59.62 | 61.02 | 60.74 | 62.72 | 61.75 |
| **Lipid (%)**^¥^ | 30.31 | 31.09 | 30.42 | 32.72 | 32.23 | 31.77 | 29.27 |

Energy, energy content of LPD in kcal per day; Protein supply, protein content of LPD as grams of protein/kg of body weight per day; Protein, protein content of LPD as a percentage of energy; Carbohydrate, carbohydrate content of LPD as a percentage of energy; Lipid, lipid content of LPD as percentage of energy.

^*^Dietary reference intake for proteins in the adult Italian population: 0.9 g/kg body weight.^41^

^§^Dietary reference intake range for carbohydrates in the adult Italian population: 45-60% of energy.^41^

^¥^Dietary reference intake range for lipids in the adult Italian population: 20-35% of energy.^41^

**Table S5.** Sequence of the primers used for quantitative RT-PCR.

| Gene | Primer sequence | | Amplicon length |
| --- | --- | --- | --- |
| *MAP1LC3B* | Fw | 5'-TTCGAGAGCAGCATCCAACC-3' | 154 bp |
|  | Rv | 5'-TTGAGCTGTAAGCGCCTTCT-3' |  |
| *BECN1* | Fw | 5'-TGGAAGGGTCTAAGACGT-3' | 149 bp |
|  | Rv | 5'-GGCTGTGGTAAGTAATGGA-3' |  |
| *GAPDH* | Fw | 5'-GTCAAGGCTGAGAACGGGAA-3' | 158 bp |
|  | Rv | 5'-AAATGAGCCCCAGCCTTCTC-3' |  |
